# Identification of HLA-A*02*:06:01* as the primary disease susceptibility HLA allele in cold medicine-related Stevens-Johnson syndrome with severe ocular complications by high-resolution NGS-based HLA typing

**DOI:** 10.1038/s41598-019-52619-2

**Published:** 2019-11-07

**Authors:** Ken Nakatani, Mayumi Ueta, Seik-Soon Khor, Yuki Hitomi, Yuko Okudaira, Anri Masuya, Yuki Wada, Chie Sotozono, Shigeru Kinoshita, Hidetoshi Inoko, Katsushi Tokunaga

**Affiliations:** 1 Department of Human Genetics, Graduate School of Medicine, The University of Tokyo, Tokyo, Japan; 2 Department of Frontier Medical Science and Technology for Ophthalmology, Kyoto Prefectural University of Medicine, Kyoto, Japan; 3 GenoDive Pharma Inc., Kanagawa, Japan; 4 The Center of Medical Innovation and Translational Research, Graduate School of Medicine, Osaka University, Osaka, Japan; 5 Department of Ophthalmology, Kyoto Prefectural University of Medicine, Kyoto, Japan

**Keywords:** Genetic association study, Disease genetics

## Abstract

Stevens-Johnson syndrome (SJS) and toxic epidermal necrolysis (TEN) are life-threatening acute inflammatory vesiculobullous reactions of the skin and mucous membranes. These severe cutaneous drug reactions are known to be caused by inciting drugs and infectious agents. Previously, we have reported the association of HLA-A*02:06 and HLA-B*44:03 with cold medicine (CM)-related SJS/TEN with severe ocular complications (SOCs) in the Japanese population. However, the conventional HLA typing method (PCR-SSOP) sometimes has ambiguity in the final HLA allele determination. In this study, we performed HLA-disease association studies in CM-SJS/TEN with SOCs at 3- or 4-field level. 120 CM-SJS/TEN patients with SOCs and 817 Japanese healthy controls are HLA genotyped using the high-resolution next-generation sequencing (NGS)-based HLA typing of HLA class I genes, including *HLA-A*, *HLA-B*, and *HLA-C*. Among the alleles of HLA class I genes, HLA-A*02:06:01 was strongly associated with susceptibility to CM-SJS/TEN (*p* = 1.15 × 10^−18^, odds ratio = 5.46). Four other alleles (HLA-A*24:02:01, HLA-B*52:01:01, HLA-B*46:01:01, and HLA-C*12:02:02) also demonstrated significant associations. HLA haplotype analyses indicated that HLA-A*02:06:01 is primarily associated with susceptibility to CM-SJS/TEN with SOCs. Notably, there were no specific disease-causing rare variants among the high-risk HLA alleles. This study highlights the importance of higher resolution HLA typing in the study of disease susceptibility, which may help to elucidate the pathogenesis of CM-SJS/TEN with SOCs.

## Introduction

Stevens-Johnson syndrome (SJS) and its severe condition, toxic epidermal necrolysis (TEN), are acute inflammatory vesiculobullous reactions of the skin, mucous membranes, genitals, and eyes. These reactions are rare, with the annual incidence of SJS and TEN reportedly 1–6 and 0.4–1.2 cases per million persons, respectively^1,2^ and a mortality rate of 3% and 27%, respectively^3^. SJS and TEN are often associated with inciting drugs and infectious agents^4–6^.

It has been reported that specific HLA genotypes are associated with drug-induced severe cutaneous adverse reactions (SCARs), including SJS/TEN. Carbamazepine-induced SJS/TEN is reportedly associated with HLA-B*15:02 in Taiwanese Han Chinese patients^7^ and with HLA-A*31:01 in Japanese^8^ and European individuals^9^. Allopurinol which commonly used to treat gout and specific types of kidney stones, also induces SCARs, including SJS/TEN, and allopurinol-induced SCARs are previously reported to be strongly associated with HLA-B* 58:01 in Han Chinese^10^, Caucasian^11^, and Japanese patients^12^. In addition to allopurinol and carbamazepine, cold medicines, including nonsteroidal anti-inflammatory drugs (NSAIDs) and multi-ingredient medications are also major causative agents for SJS/TEN^1,5^. Our group previously reported that in the Japanese population, cold medicine-related SJS/TEN (CM-SJS/TEN) with severe ocular complications (SOCs) is strongly associated with HLA-A*02:06 (cases, n = 151; controls, n = 639; *p* = 2.7 × 10^−20^; odds ratio [OR] = 5.6) and HLA-B*44:03 (cases, n = 151; controls, n = 639; *p* = 1.3 × 10^−3^; OR = 2.0)^13^.

Currently, HLA allele typing is primarily performed using two conventional typing methods, PCR sequence-specific oligonucleotide probing (PCR-SSOP) (e.g., Luminex methodology) and PCR sequence-based typing (SBT)^14,15^. However, these conventional typing methods are designed for targeting only key exons (exons 2 and 3) in HLA class I genes in which polymorphisms are known to be functionally relevant to HLA diversity. This limited target region presents difficulties in terms of final HLA allele assignment in cases in which a number of possible alleles remain as ambiguity alleles; in addition, resolution of only up to 2-field (often referred to as 4-digit) can be achieved.

In recent years, several next-generation sequencing (NGS) based HLA typing methods using have been developed^16–20^. NGS-based HLA typing methods enable the determination of the entire HLA gene sequence for HLA class I genes. Moreover, novel polymorphisms that may be present in non-key exons, introns, and untranslated regions (UTRs) can be determined. Due to the high-resolution sequencing possible with NGS-based HLA typing, HLA allele typing at 3- or 4-field (6- or 8-digit) resolution can be achieved, in addition to resolving HLA ambiguities seen with conventional HLA allele typing methods. In addition, several studies have shown that single nucleotide polymorphisms/variations (SNPs/SNVs) affecting the expression level of HLA genes exist in the promoter region^21^ or 3′UTR^22^ of HLA genes; thus, 3- or 4-field HLA allele typing may be important in disease susceptibility studies.

In this study, we explored possible associations between HLA 3- or 4-field genotypes and CM-SJS/TEN with SOCs using super-high-resolution NGS-based HLA typing.

## Methods

### Patients and controls

Samples from 120 Japanese patients with CM-SJS/TEN with SOCs were collected at Kyoto Prefectural University of Medicine (KPUM). The subjects included 48 males and 72 females, ranging in age from 6 to 85 years (median age 38 ± 17.1 [SD] years). Age at SJS/TEN onset ranged from 1 to 76 years (median age 23.5 ± 16.1 [SD] years). The diagnosis of SJS/TEN with SOCs was based on a confirmed history of acute-onset high fever, serious mucocutaneous illness with skin eruptions, and the involvement of at least 2 mucosal sites, including the oral cavity and ocular surface. All CM-SJS/TEN patients included in the current study had taken cold medicines (e.g., NSAIDs or multi-ingredient cold medications) several days before disease onset. The specific drugs used are listed in Supplementary Table 1, although not all specific drugs consumed by the patients were identified.

Healthy Japanese volunteers (n = 817) served as the controls. They were independently recruited by the University of Tokyo (n = 419; 350 females, 69 males; ages unknown) and Pharma SNP Consortium (n = 398; 106 females, 292 males; median age 47.5 ± 11.8 [SD] years).

Genomic DNA was isolated at SRL Inc. (Tokyo, Japan) from peripheral blood collected at KPUM. For Japanese control samples from the Pharma SNP Consortium, genomic DNA was extracted from peripheral blood leukocytes using standard techniques. For extraction of genomic DNA from Japanese control subject peripheral blood samples collected at the University of Tokyo, a commercial kit (QIAamp Blood Kit, Qiagen, Hilden, Germany) was used.

This study was approved by the institutional review board of KPUM, Kyoto, Japan, and the Faculty of Medicine, University of Tokyo, Tokyo, Japan. The purpose of the study and the experimental protocols were explained to all patients, and their prior written informed consent was obtained. All the experiment methods were performed in accordance with University of Tokyo, Tokyo, Japan guidelines and regulations. For research involving human participants under the age of 18 years informed consent have been obtained from parents.

### HLA allele typing using NXType^™^ and AllType^™^ NGS HLA typing kits

*HLA-A*, *-B*, and *-C* alleles of all 120 patients and 817 controls were analyzed in the present study. HLA allele typing was performed using an NXType^™^ Class I NGS HLA typing kit and an AllType^™^ NGS 11-Loci Amplification kit (Thermo Fisher Scientific, Waltham, MA, USA).

Briefly, sample DNA was amplified using the long-range multiplex primers designed for the HLA class I genes. Amplicon concentrations were measured using Qubit 2.0 (Thermo Fisher Scientific) with the Qubit dsHS reagent kit according to the manufacturer’s protocol. Using the multiplex PCR amplicons, library preparation was carried out using an Ion Xpress^™^ Plus Fragment Library kit (Thermo Fisher Scientific) and Ion Xpress^™^ Shear Plus kit (Thermo Fisher Scientific). Barcode ligation was carried out using an Ion Xpress^™^ Barcode Adaptors 1–96 kit (Thermo Fisher Scientific). DNA sizing and quantitation for each library were carried out using an Agilent 2100 Expert Bioanalyzer with an Agilent High Sensitivity DNA kit (Agilent Technologies, Santa Clara, CA, USA). Purification of DNA fragments during library preparation was performed using a Beckman Ampure XP system (Beckman Coulter, Inc., Brea, CA, USA). For the NXType kit, isothermal amplification (IA) was performed using an Ion PGM^™^ Template IA 500 reagents kit (Thermo Fisher Scientific). Beads carrying the single-stranded DNA templates were enriched using a OneTouch^™^ ES instrument (Thermo Fisher Scientific). Sequencing was performed using an Ion PGM^™^ Sequencing Hi-Q^™^ kit and 318 Chip kit v2 (Thermo Fisher Scientific). For the AllType kit, IA and enrichment steps were carried out using an Ion S5^™^ ExT Chef kit (Thermo Fisher Scientific) and Ion Chef^™^ instrument (Thermo Fisher Scientific), and sequencing was performed using Ion S5^™^ sequencing reagents (Thermo Fisher Scientific) and an Ion 530^™^ chip (Thermo Fisher Scientific).

After the sequencing run, all raw data were automatically saved in the Ion Torrent Server and converted to sequence fastq files for each sample. These fastq files were then analyzed, and HLA allele calling was performed using TypeStream^™^ Visual NGS Analysis software, v.1.1 Hot Fix 1, with the IMGT/HLA 3.29.0 databases.

### Validation by sanger sequencing

In order to validate Luminex-discordant, null, and newly discovered alleles, purified PCR amplicons were sequenced using a BigDye^™^ Terminator kit v.3.1 (Thermo Fisher Scientific) and 3730xl DNA Analyzer (Thermo Fisher Scientific). The primers (Supplementary Table 3) were designed according to the genomic sequences of candidate regions using Primer-BLAST^23^. The generated chromatogram sequence data were analyzed using BioEdit software, v.7.0.5 (http://www.mbio.ncsu.edu/BioEdit/bioedit.html).

### HLA haplotype estimation

Each HLA class I gene haplotype was estimated using BIGDAWG (Bridging ImmunoGenomic Data-Analysis Workflow Gaps) software, version 2.1, implemented as the bigdawg R package^24^.

### Statistical analysis

The carrier frequencies of individual HLA alleles in patients and controls were compared based on the dominant model using the *χ*^*2*^-test (R software; R Foundation for Statistical Computing). HLA alleles and haplotypes with frequencies less than 1% in cases and controls were excluded from the association analysis. Fisher’s exact test (R software; R Foundation for Statistical Computing) was used when one or more observed counts was less than 5. Significance levels were corrected by Bonferroni correction for multiplicity of testing by the number of comparisons. A corrected *P* value of <0.05 was considered statistically significant.

## Results

### NGS-based HLA allele typing

A total of 95 HLA class I allele sequences (23 *HLA-A*, 47 *HLA-B*, 25 *HLA-C*) at the 3-field level were detected for all SJS cases and healthy controls using NGS-based HLA allele typing (Table 1, Supplementary Table 2). For cases in which 3-field level sequences were not registered in the IMGT/HLA database v.3.29.0, 2-field (4-digit) allele assignments (such as C*14:03) were adopted. Newly discovered alleles were validated by Sanger sequencing, and all alleles were correctly sequenced by NGS (Supplementary Fig. 1a–d). The number of alleles subdivided into multiple 3-field alleles is summarized in Table 2.

**Table 1:** Characterization of HLA class I alleles determined by NGS-based HLA typing of 937 samples.

Locus	Number of HLA alleles (2-field)	Number of HLA alleles (3-field)	Number of detected novel allele (3-field)	Novel alleles/all alleles	Number of detected null alleles
A	22	23	1	0.053%	1
B	42	47	1	0.053%	0
C	21	25	2	0.11%	0
total	85	95	4	0.21%	1

**Table 2:** Number of HLA class I alleles subdivided into multiple 3-field-level alleles (2n = 1,874).

HLA alleles (2-field)	Number of HLA alleles (2-field)	HLA alleles (3-field)	Number of HLA alleles (3-field)
A*31:01	159	A*31:01:NEW	1
		A*31:01:02	158
B*39:01	77	B*39:01:01	31
		B*39:01:03	46
B*39:02	5	B*39:02:01	4
		B*39:02:03	1
B*44:03	176	B*44:03:01	175
		B*44:03:02	1
B*48:01	57	B*48:01:01	56
		B*48:01:NEW	1
B*67:01	19	B*67:01:01	16
		B*67:01:02	3
C*03:03	241	C*03:03:01	239
		C*03:03:17	1
		C*03:03:NEW	1
C*03:04	238	C*03:04:01	237
		C*03:04:04	1
C*14:03	176	C*14:03	175
		C*14:03:NEW	1

For some samples (SJS, n = 105; controls, n = 752), 2-field Luminex HLA allele typing results were available from our previous study. The accuracy of NGS-based HLA allele typing was evaluated by assessing the concordance between Luminex-based HLA typing results and NGS-based HLA allele typing results at 2-field resolution. Seven discordant alleles were observed, as shown in Table 3. Additional Sanger sequencing was performed to evaluate the key nucleotide differences between the results of NGS-based HLA allele typing and Luminex-based HLA allele typing, which demonstrated that the NGS results were accurate for these 7 alleles (Supplementary Fig. 1e–k). For example, A*02:15N has been detected at an allele frequency of 0.003% in the Japanese population^25^, and the differences in the exon sequences between A*02:15 N and A*02:07 exist in exon 4 of the *HLA-A* gene, which is not covered by Luminex oligonucleotide probes. Therefore, these two alleles are listed among Luminex HLA ambiguities.

**Table 3:** Discordance between NGS typing and Luminex typing results (SJS: n = 105, controls: n = 752).

NGS typing	PCR-SSOP (Luminex) typing	N	Discordant base position	Luminex probe
A*02:15N	A*02:07	1	Exon 4 (1 base)	out of target
A*11:02	A*11:01	1	Exon 2 (2 bases)	✓(Typing error)
B*07:169	B*07:02	1	Exon 4 (1 base)	out of target
B*59:04	B*59:01	1	Exon 3 (2 bases)	no probe
C*04:82	C*04:01	14	Exon 5 (9 bases)	out of target
C*07:06	C*07:01	1	Exon 5,6 (6 bases)	out of target
C*08:22	C*08:01	7	Exon 6 (1 base)	out of target

Abbreviations: PCR-SSOP, polymerase chain reaction sequence specific oligonucleotide probing; NGS, next generation sequencing.

Discordant base position between NGS typing and Luminex typing is shown with the number of bases. “out of target” means the Luminex probe doesn’t cover the targeted reigion (exon 2 and 3). “no probe” means Luminex probe doesn’t cover the discordant base position.

“✓(Typing error)” means the Luminex probe covers the discordant base position, but can’t distinguish the difference because of some typing error.

### Significant associations between HLA alleles and susceptibility to CM-SJS/TEN with SOCs

Carrier frequencies of HLA alleles were compared between 120 CM-SJS/TEN with SOCs patients and 817 healthy controls. We compared 42 alleles (10 for *HLA-A*, 18 for *HLA-B*, 14 for *HLA-C*) carried by more than 1% of individuals among both patients and controls. In total, 16 alleles (6 for *HLA-A*, 6 for *HLA-B*, 4 for *HLA-C*) demonstrated associations with *P* values < 0.05, for which the associations of the 5 HLA alleles remained significant after correction for multiple-testing (Table 4). HLA-A*02:06:01 was strongly associated with CM-SJS/TEN with SOCs (*P* = 1.15 × 10^−18^, *Pc* = 1.15 × 10^−17^, OR = 5.46). Four alleles of HLA class I genes (*HLA-A*, *HLA-B*, and *HLA-C*) exhibited new associations; the frequency of HLA-B*46:01:01 was significantly increased in CM-SJS/TEN with SOCs (*P* = 0.0014, *Pc* = 0.0246, OR = 2.34), whereas the frequencies of HLA-A*24:02:01 (*P* = 7.81 × 10^−5^, *Pc* = 7.81 × 10^−4^, OR = 0.46), HLA-B*52:01:01 (*P* = 5.90 × 10^−4^, *Pc* = 0.0106, OR = 0.33), and HLA-C*12:02:02 (*P* = 0.0013, *Pc* = 0.0181, OR = 0.36) were significantly decreased in CM-SJS/TEN with SOCs.

**Table 4:** Carrier frequencies of HLA class I alleles in CM-SJS/TEN patients and healthy controls.

HLA alleles/haplotypes	Carrier frequency (%)				Dominant model association analysis			
	Case (n = 120) (%)		Control (n = 817) (%)		*P*	*P-corrected*	Odds ratio (95%CI)	
*HLA-A*								
A*02:06:01	59	(49.2%)	123	(15.1%)	1.15.E-18	1.15.E-17	5.46	(3.64–8.19)
A*24:02:01	50	(41.7%)	496	(60.7%)	7.81.E-05	7.81.E-04	0.46	(0.31–0.68)
*HLA-B*								
B*46:01:01	21	(17.5%)	68	(8.3%)	0.0014	0.0246	2.34	(1.37–3.98)
B*52:01:01	10	(8.3%)	178	(21.8%)	5.90.E-04	0.0106	0.33	(0.17–0.64)
*HLA-C*								
C*12:02:02	11	(9.2%)	178	(21.8%)	0.0013	0.0181	0.36	(0.19–0.69)
*A-B*								
A*02:06:01-B*35:01:01	14	(11.7%)	23	(2.8%)	7.11.E-05	2.28.E-03	4.55	(2.1–9.55)
A*24:02:01-B*52:01:01	10	(8.3%)	169	(20.7%)	0.0013	0.0419	0.35	(0.18–0.68)
*B-C*								
B*46:01:01-C*01:02:01	20	(16.7%)	58	(7.1%)	3.96.E-04	0.0099	2.62	(1.51–4.53)
B*52:01:01-C*12:02:02	10	(8.3%)	175	(21.4%)	7.72.E-04	0.0193	0.33	(0.17–0.65)
*C-A*								
A*02:06:01-C*01:02:01	12	(10%)	12	(1.5%)	8.13.E-06	2.19.E-04	7.43	(2.97–18.57)
A*02:06:01-C*03:03:01	12	(10%)	23	(2.8%)	7.13.E-04	0.0193	3.83	(1.68–8.29)
A*02:06:01-C*03:04:01	11	(9.2%)	19	(2.3%)	6.27.E-04	0.0169	4.23	(1.77–9.65)

Abbreviations: HLA, human leukocyte antigen; CM-SJS/TEN, cold medicine-related Stevens-Johnson syndrome/toxic epidermal necrolysis; CI, Confidence interval.

HLA alleles and haplotypes with frequencies < 1% in cases and controls are excluded from the association analysis. *P-corrected*: *P* values for allele/haplotype frequency comparisons between cases and controls using the Peason’s chi-square test or Fisher’s exact test and then corrected for the multiplicity of testing by the number of comparisons. (10, 18, and 14 for HLA-A, HLA-B, and HLA-C, respectively. 32, 25, and 27 for A-B, B-C, and C-A haplotype, respectively).

### HLA haplotype association analyses

In order to identify the primary associations among HLA class I alleles, haplotype analyses were conducted using BIGDAWG software. We compared 84 haplotypes (32 for A-B, 25 for B-C, 27 for A-C haplotypes) carried by more than 1% of individuals among both patients and controls. Twenty haplotypes (5 for A-B, 7 for B-C, 8 for A-C haplotypes) demonstrated associations with *P* values < 0.05, and the associations of 7 haplotypes remained significant after corrected for multiple-testing (Table 4). The frequencies of HLA-A*02:06:01-HLA-B*35:01:01 (*Pc* = 2.28 × 10^−3^, OR = 4.55), HLA-B*46:01:01-HLA-C*01:02:01 (*Pc* = 0.0099, OR = 2.62), HLA-A*02:06:01-HLA-C*01:02:01 (*Pc* = 2.19 × 10^−4^, OR = 7.43), HLA-A*02:06:01-HLA-C*03:03:01 (*Pc* = 0.0193, OR = 3.83), and HLA-A*02:06:01-HLA-C*03:04:01 (*Pc* = 0.0169, OR = 4.23) were significantly increased in CM-SJS/TEN with SOCs. In contrast, the frequencies of HLA-A*24:02:01-HLA-B*52:01:01 (*Pc* = 0.0419, OR = 0.35) and HLA-B*52:01:01-HLA-C*12:02:02 (*Pc* = 0.0193, OR = 0.33) were significantly decreased in CM-SJS/TEN with SOCs.

## Discussion

In this study, we investigated potential novel HLA alleles associated with the occurrence of CM-SJS/TEN with SOCs using NGS-based high-resolution HLA allele typing. To our knowledge, there have been no reports published to date regarding case-control association analyses of HLA alleles at the 3-field level using NGS-based HLA typing. Our approach enabled 3-field HLA allele assignment in which previous associations could be analyzed at higher resolution. In addition, this approach provided full resolution of ambiguous HLA alleles in comparison with traditional HLA allele typing methods such as PCR-SSOP or PCR-SBT. This could ultimately provide a more detailed picture of the relationship between HLA polymorphism profiles and disease susceptibility.

Our group previously reported that HLA-A*02:06 was strongly associated with susceptibility to CM-SJS/TEN with SOCs^13^. In this study, HLA-A*02:06:01 showed the strongest association at the 3-field level, and all previously typed HLA-A*02:06 alleles were classified as HLA-A*02:06:01 at the 3-field level. A previous study using *in silico* docking simulations reported that the protein encoded by HLA-A*02:06 is predicted to bind to various ingredients contained in cold medicines, such as acetaminophen, although the predicted binding was not verified experimentally^26^. These findings suggest that the association of HLA-A*02:06:01 is mainly attributed to the 2-field rather than 3-field level; that is, the differences in the amino acid sequences might be directly correlated with CM-SJS/TEN through peptide presentation. A well-known example of this phenomenon involves the specific binding of abacavir, an anti-HIV drug, with the protein encoded by HLA-B*57:01. Abacavir binds with exquisite specificity to the HLA-B*57:01–encoded protein, altering immunologic ‘self’ with the selection of new endogenous peptides^27,28^. This molecular mechanism is one possibility, but whether such reactions occur between the ingredients of cold medicines and protein product of HLA-A*02:06:01 remains unclear. Considering that the onset of SJS/TEN with SOCs is associated not only with the administration of drugs but also with viral and microbial infections, these factors might interact with each other, leading to alterations of the immune system and subsequent destruction of target cells.

In contrast, B*44:03, which our group previously reported as an independent risk factor in the Japanese population^13^, can be divided into two alleles (HLA-B*44:03:01 and HLA-B*44:03:02) at the 3-field level. Although the case group had a higher frequency of B*44:03:01 than controls, the association of B*44:03:01 did not remain significant after correction for multiple-testing, probably because of the smaller sample size of that study. However, HLA-B*44:03 appears to be a universal marker of CM-SJS in many populations, including Indian^29^, Brazilian^30^, and Thai populations^31^. Reports from the USA^32^ and France^33^ indicated that levels of the HLA-B12 (HLA-Bw44) antigen, primarily encoded by HLA-B*44:02 or HLA-B*44:03, are significantly increased in Caucasian SJS patients who had taken NSAIDs as cold medicines. Considering the possibility that the HLA-B*44:03 association can be attributed to specific ingredients in cold medicines, stratification of the case group based on drug ingredients should be examined in future studies. In this study, however, the specific drugs were not known in all patients, so further analyses targeting specific drugs are necessary.

In this study, four HLA class I alleles other than HLA-A*02:06:01 that exhibited new associations with CM-SJS/TEN with SOCs were identified. However, haplotype analyses suggested that the associations of *HLA-B* and *HLA-C* were due primarily to strong linkage disequilibrium with significant *HLA-A* alleles. The haplotype HLA-B*52:01:01- HLA-C*12:02:02 forms a common haplotype with HLA-A*24:02:01 in Japanese individuals, and the direction of the odds ratio is the same as that of HLA-A*24:02:01. These observations suggest that the HLA-B*52:01:01 and HLA-C*12:02:02 associations are not primary causative associations but rather the result of linkage disequilibrium with HLA-A*24:02:01. Similar haplotype associations were also observed for HLA-B*46:01:01, which forms a common haplotype with HLA-C*01:02:01 and HLA-A*02:06:01, and the direction of the odds ratio is the same as that of HLA-A*02:06:01, suggesting that the HLA-B*46:01:01 association can be attributed to the effect of HLA-A*02:06:01. In addition, HLA-A*24:02:01, which exhibited a protective association, is the most frequent *HLA-A* allele in the Japanese population (the allele frequency of HLA-A*24:02 is 36.1%)^25^. Thus, it is possible that the increase in HLA-A*02:06:01 in the case group caused the decrease in HLA-A*24:02:01, leading to the apparent protective association of HLA-A*24:02:01. In order to evaluate the susceptible and protection effect of HLA-A*02:06:01 and HLA-A*24:02:01, we compared the frequencies of CM-SJS/TEN with SOCs patients and healthy controls carrying both HLA-A*02:06:01 and HLA-A*24:02:01. The percentage of CM-SJS/TEN with SOCs patients and controls carrying both HLA alleles are 11.7% and 5.01% respectively, indicating that the susceptible allele effect of HLA-A*02:06:01 is stronger than the apparent protective effect of HLA-A*24:02:01.

Notably, NGS of HLA class I genes revealed no specific disease-causing rare variants among the high-risk HLA alleles; that is, HLA genes contribute to the susceptibility to CM-SJS/TEN with SOCs. In previous studies, several immune-related genes other than *HLA* have been linked to susceptibility to CM-SJS/TEN with SOCs, such as *PTGER3*^34^, *IKZF1*^35^, *TSHZ2*^35^, *IL-4R*^36^, *FasL*^37^, *IL-13*^38^, and *TLR3*^4,34^, suggesting that the combination of multiple gene polymorphisms and their interactions, including with HLA alleles, contributes strongly to the onset of CM-SJS/TEN with SOCs. This hypothesis is supported in part by the findings of this study indicating that HLA genes are associated with disease susceptibility rather causality.

Although almost all of 3-field alleles subdivided from the same 2-field alleles belonged to one of the alleles (Table 2); some alleles were divided into 2 subgroups with substantial frequencies (e.g., 77 B*39:01 was divided into 31 B*39:01:01 and 46 B*39:01:03). Although peptide presentation possibly affects disease susceptibility, it has been reported that coding variants affecting regions outside of the peptide-binding groove are strongly associated with diseases such as type 1 diabetes^39^. These data support the hypothesis that higher-resolution NGS-based HLA allele typing is useful for disease association studies. In addition, one of the objectives of this study was to investigate the impact of non-coding variants on disease susceptibility. However, we were only able to achieve 3-field level HLA allele assignment and could not determine assignments at the 4-field level for some of the samples. This limitation occurred due to 2 major factors: (i) lack of amplification of the UTR by the NXType kit for several samples, and (ii) insufficient 4-field HLA allele sequences in the current IMGT/HLA database. Several studies have shown that HLA non-coding SNPs are associated with diseases, such as an association of an SNP in the 3′UTR of *HLA-DPB1* with acute graft-versus-host disease^22^ and of a specific SNP in intron 2 of *HLA-DRB1* with rheumatoid arthritis^40^. Therefore, in order to consider the effect of non-coding variants on disease susceptibility, technical improvements in reagents and/or typing software are needed, in addition to an expansion of 4-field HLA reference sequences in public databases.

In conclusion, although some technical limitations have to be noted, we successfully identified HLA class I alleles at the 3-field level using high-resolution NGS-based HLA allele typing and demonstrated a strong association between CM-SJS/TEN with SOCs and HLA-A*02:06:01 in the Japanese population. These findings highlight the importance of higher-resolution HLA typing in the study of disease susceptibility, which may help to elucidate the pathogenesis of CM-SJS/TEN with SOCs.

## Supplementary information

Supplementary Figure 1a-k, Supplementary Table 1, Supplementary Table 2, Supplementary Table 3
