# Supplementary material for: Identification of HLA-A*02:06:01 as the primary disease susceptibility HLA allele in cold medicine-related Stevens-Johnson syndrome with severe ocular complications by high-resolution NGS-based HLA typing
Source: Sci Rep. 2019 Nov 7;9:16240. doi: 10.1038/s41598-019-52619-2 (PMC6838058; doi:10.1038/s41598-019-52619-2)
Supplement: Supplementary file 1 — Supplementary Figure 1a-k, Supplementary Table 1, Supplementary Table 2, Supplementary Table 3 [file 41598_2019_52619_MOESM1_ESM.docx]

**Identification of *HLA-A*02:06:01* as the primary disease susceptibility HLA allele in cold medicine-related Stevens-Johnson syndrome with severe ocular complications by high-resolution NGS-based HLA typing**

Ken Nakatani^1,6^, Mayumi Ueta^2,6^, Seik-Soon Khor^1^, Yuki Hitomi^1^, Yuko Okudaira^3^, Anri Masuya^3^, Yuki Wada^4^, Chie Sotozono^5^, Shigeru Kinoshita^2^, Hidetoshi Inoko^3^, Katsushi Tokunaga^1^

1. Department of Human Genetics, Graduate School of Medicine, The University of Tokyo, Tokyo, Japan
2. Department of Frontier Medical Science and Technology for Ophthalmology, Kyoto Prefectural University of Medicine, Kyoto, Japan
3. GenoDive Pharma Inc., Kanagawa, Japan
4. The Center of Medical Innovation and Translational Research, Graduate School of Medicine, Osaka University, Osaka, Japan
5. Department of Ophthalmology, Kyoto Prefectural University of Medicine, Kyoto, Japan
6. These authors contributed equally.

**Supplemental material**

**
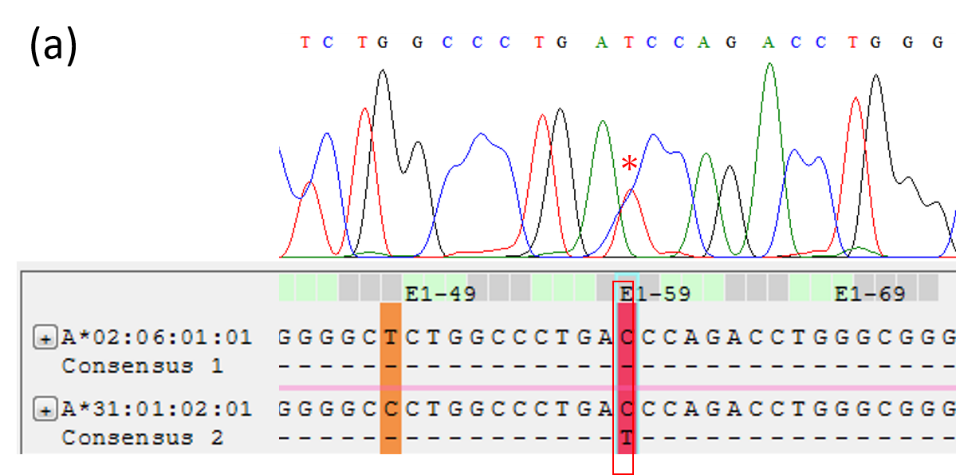
**

**
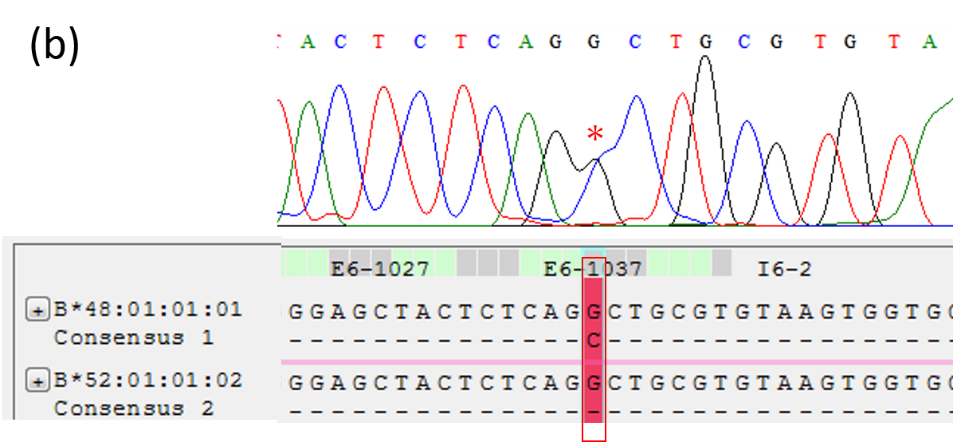

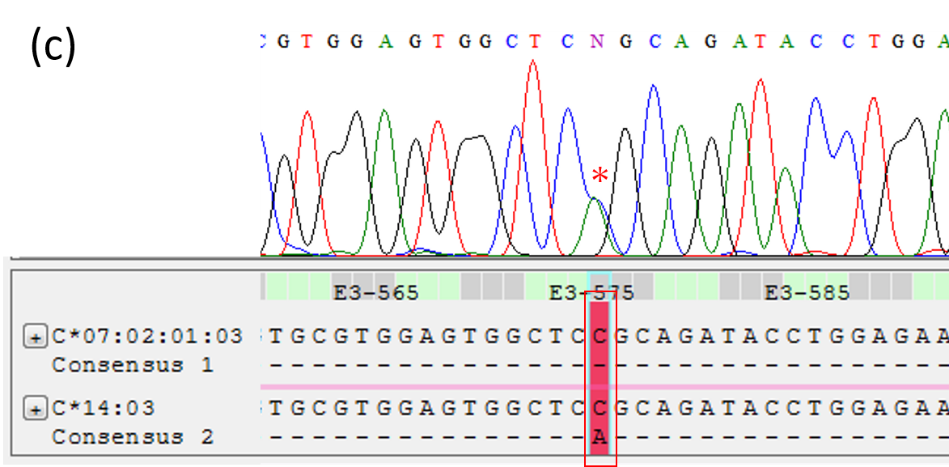
**

**
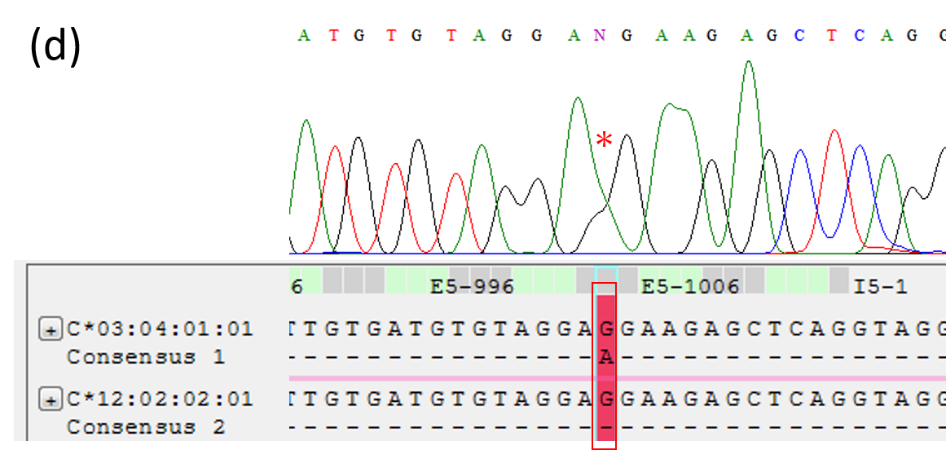
**

**
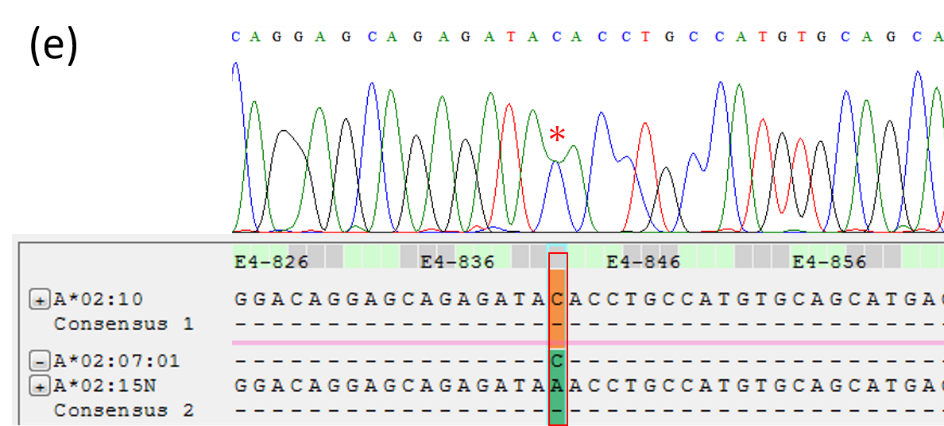
**

**
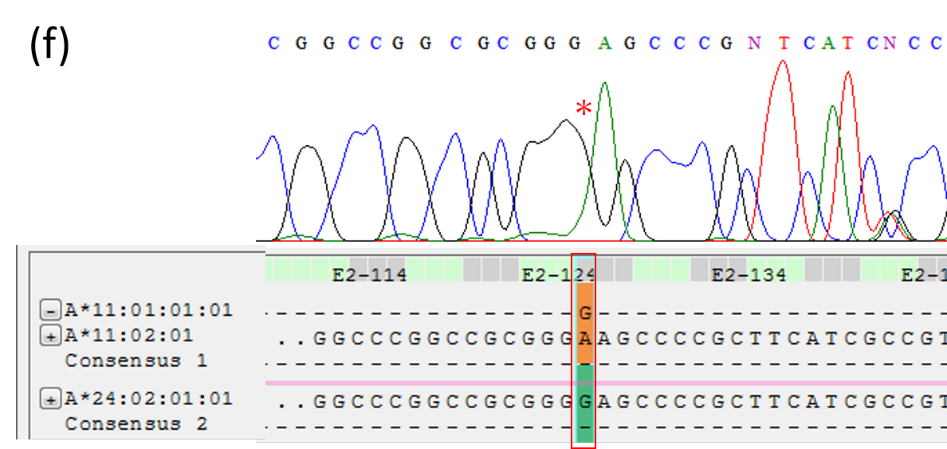
**

**
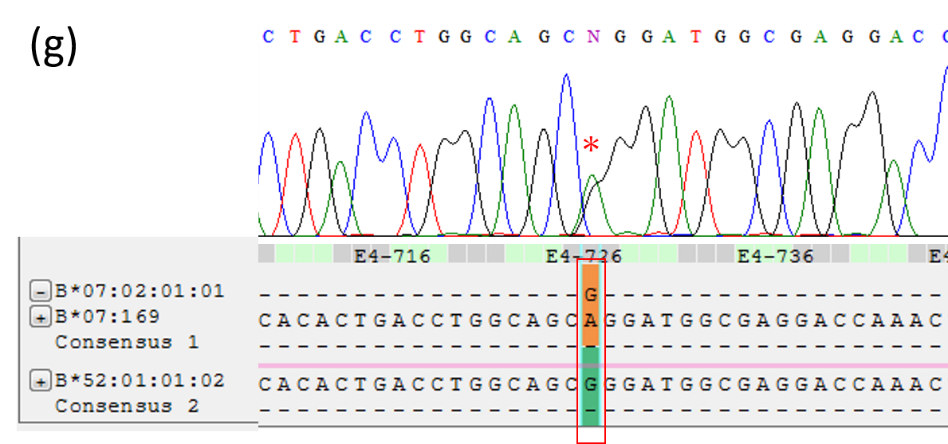
**

**
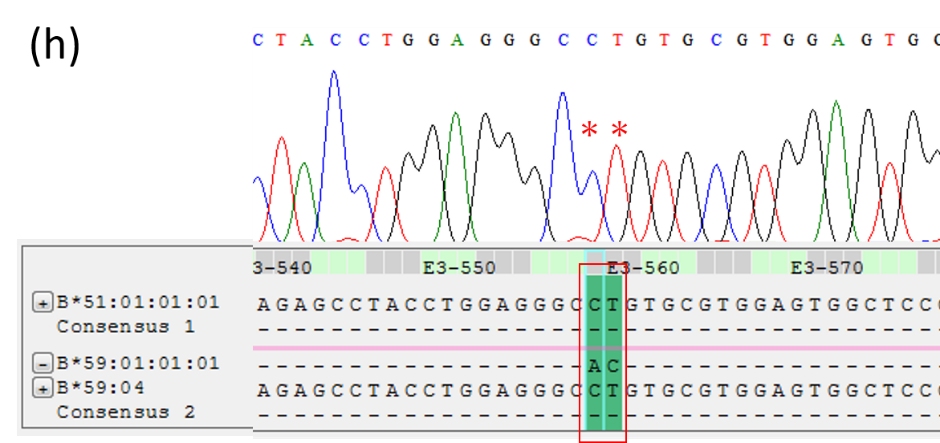
**

**
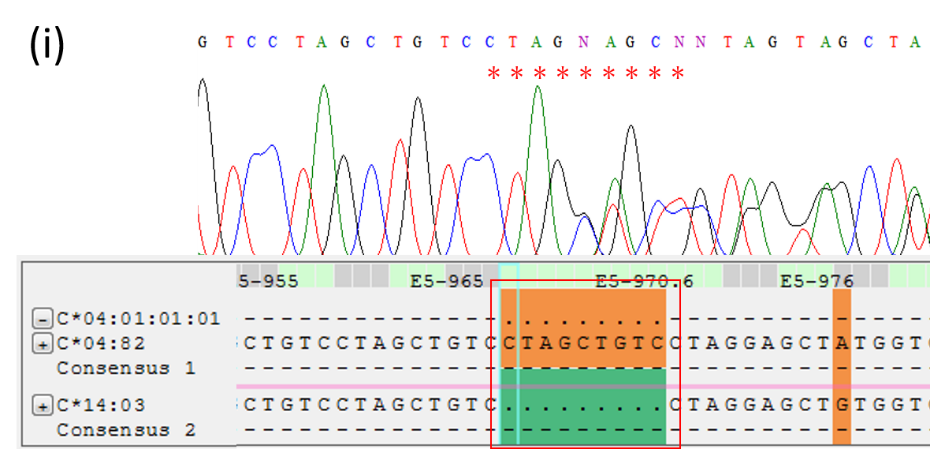
**

**
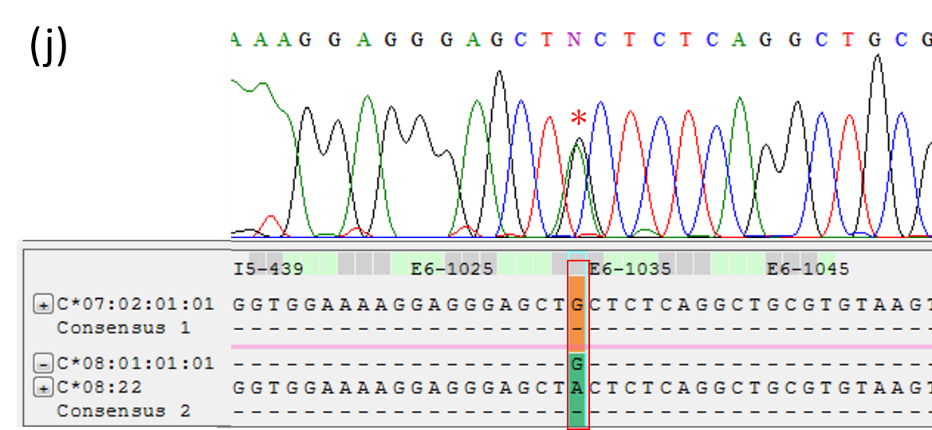
**

**
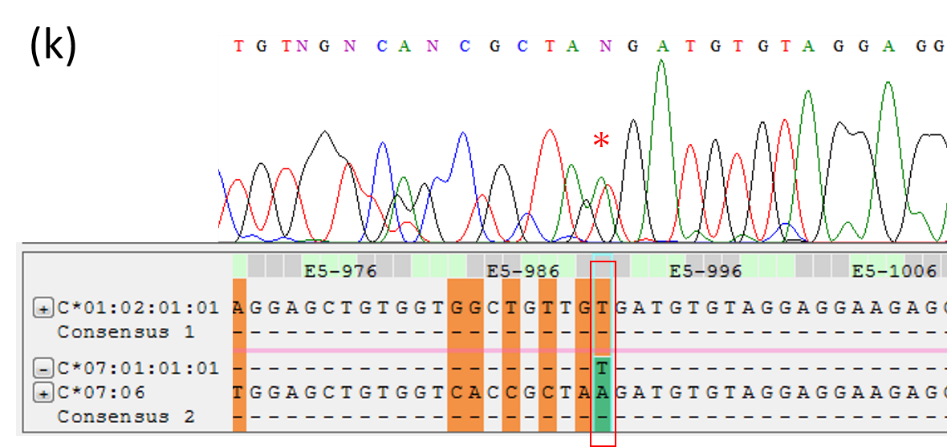
**

**Supplementary Figure 1a-k |　Results of Sanger sequencing and Nucleotide Sequence Viewer of the identified mutation locus in TypeStream Visual software.**

The consensus sequence is displayed for each allele next to the sequence for the reference allele. Every matched position displays as a dash in the consensus sequence. **(a-d)** Newly discovered alleles. **(a)** *HLA-A*31:01:NEW*, HLA-A c.59C>T, **(b)** *HLA-B*48:01:NEW*, HLA-B c.1039G>C, **(c)** *HLA-C*14:03:NEW*, HLA-C c.577C>A, **(d)** *HLA-C*03:04:NEW*, HLA-C c.1004G>A, and **(e-k)** Luminex discordant alleles. The reference sequence for the Luminex typing allele is compared with the corresponding NGS allele. **(e)** *HLA-A*02:15N,* HLA-A c.843C>A; **(f)** *HLA-A*11:02:01,* HLA-A c.127G>A; **(g)** *HLA-B*07:169,* HLA-B c.726G>A; **(h)** *HLA-B*59:04,* HLA-B c.559A>C, HLA-B c.560C>T; **(i)** *HLA-C*04:82,* HLA-C c.970_971insCTAGCTGTC; **(j)** *HLA-C*08:22,* HLA-C c.1034G>A; **(k)** *HLA-C*07:06,* HLA-C c.992T>A, HLA-C c.1043C>T.

| **Supplementary Table 2 \| Carrier frequencies of 95 identified HLA class I alleles** | | | | | | |  |  |
| --- | --- | --- | --- | --- | --- | --- | --- | --- |
| HLA alleles | Carrier frequency (%) | | | |  | Dominant model association analysis | | |
|  | Case (n=120) (%) | | Control (n=817) (%) | |  | *P* | Odds ratio (95%CI) | |
| *HLA-A* |  |  |  |  |  |  |  |  |
| *A*02:01:01* | 32 | (26.7%) | 148 | (18.1%) |  | 0.0264 | 1.64 | (1.06-2.56) |
| *A*02:06:01* | 59 | (49.2%) | 123 | (15.1%) |  | 1.15.E-18 | 5.46 | (3.64-8.19) |
| *A*02:07:01* | 8 | (6.7%) | 46 | (5.6%) |  | - | 1.20 | (0.55-2.6) |
| *A*03:01:01* | 3 | (2.5%) | 6 | (0.7%) |  | - | 3.47 | (0.86-14.05) |
| *A*11:01:01* | 11 | (9.2%) | 139 | (17%) |  | 0.0286 | 0.49 | (0.26-0.94) |
| *A*24:02:01* | 50 | (41.7%) | 496 | (60.7%) |  | 7.81.E-05 | 0.46 | (0.31-0.68) |
| *A*24:20:01* | 1 | (0.8%) | 18 | (2.2%) |  | - | 0.37 | (0.05-2.82) |
| *A*26:01:01* | 9 | (7.5%) | 119 | (14.6%) |  | 0.0353 | 0.48 | (0.23-0.96) |
| *A*26:02:01* | 2 | (1.7%) | 28 | (3.4%) |  | - | 0.48 | (0.11-2.03) |
| *A*26:03:01* | 2 | (1.7%) | 41 | (5%) |  | - | 0.32 | (0.08-1.34) |
| *A*30:01:01* | 1 | (0.8%) | 1 | (0.1%) |  | - | 6.86 | (0.43-110.36) |
| *A*31:01:02* | 17 | (14.2%) | 134 | (16.4%) |  | - | 0.84 | (0.49-1.45) |
| *A*33:03:01* | 30 | (25%) | 138 | (16.9%) |  | 0.0306 | 1.64 | (1.04-2.58) |
| *A*01:01:01* | 0 | (0%) | 14 | (1.7%) |  | - | 0.00 |  |
| *A*02:10* | 0 | (0%) | 5 | (0.6%) |  | - | 0.00 |  |
| *A*02:15N* | 0 | (0%) | 1 | (0.1%) |  | - | 0.00 |  |
| *A*02:18* | 0 | (0%) | 1 | (0.1%) |  | - | 0.00 |  |
| *A*03:02:01* | 0 | (0%) | 2 | (0.2%) |  | - | 0.00 |  |
| *A*11:02:01* | 0 | (0%) | 3 | (0.4%) |  | - | 0.00 |  |
| *A*24:08* | 0 | (0%) | 2 | (0.2%) |  | - | 0.00 |  |
| *A*26:05* | 0 | (0%) | 3 | (0.4%) |  | - | 0.00 |  |
| *A*31:01:NEW* | 0 | (0%) | 1 | (0.1%) |  | - | 0.00 |  |
| *A*33:01:01* | 0 | (0%) | 1 | (0.1%) |  | - | 0.00 |  |
| *HLA-B* |  |  |  |  |  |  |  |  |
| *B*07:02:01* | 10 | (8.3%) | 101 | (12.4%) |  | - | 0.64 | (0.33-1.27) |
| *B*13:01:01* | 8 | (6.7%) | 21 | (2.6%) |  | 0.0155 | 2.71 | (1.17-6.26) |
| *B*13:02:01* | 1 | (0.8%) | 2 | (0.2%) |  | - | 3.42 | (0.31-38.06) |
| *B*15:01:01* | 10 | (8.3%) | 139 | (17%) |  | 0.0152 | 0.44 | (0.23-0.87) |
| *B*15:11:01* | 1 | (0.8%) | 11 | (1.3%) |  | - | 0.62 | (0.08-4.81) |
| *B*15:18:01* | 5 | (4.2%) | 20 | (2.4%) |  | - | 1.73 | (0.64-4.71) |
| *B*15:27:01* | 1 | (0.8%) | 2 | (0.2%) |  | - | 3.42 | (0.31-38.06) |
| *B*27:04:01* | 1 | (0.8%) | 3 | (0.4%) |  | - | 2.28 | (0.24-22.1) |
| *B*35:01:01* | 22 | (18.3%) | 116 | (14.2%) |  | - | 1.36 | (0.82-2.24) |
| *B*37:01:01* | 1 | (0.8%) | 11 | (1.3%) |  | - | 0.62 | (0.08-4.81) |
| *B*39:01:01* | 3 | (2.5%) | 28 | (3.4%) |  | - | 0.72 | (0.22-2.41) |
| *B*39:01:03* | 6 | (5%) | 40 | (4.9%) |  | - | 1.02 | (0.42-2.47) |
| *B*39:02:01* | 1 | (0.8%) | 3 | (0.4%) |  | - | 2.28 | (0.24-22.1) |
| *B*40:01:02* | 23 | (19.2%) | 86 | (10.5%) |  | 0.0058 | 2.02 | (1.21-3.34) |
| *B*40:02:01* | 16 | (13.3%) | 111 | (13.6%) |  | - | 0.98 | (0.56-1.72) |
| *B*40:06:01* | 6 | (5%) | 70 | (8.6%) |  | - | 0.56 | (0.24-1.32) |
| *B*44:02:01* | 3 | (2.5%) | 7 | (0.9%) |  | - | 2.97 | (0.76-11.63) |
| *B*44:03:01* | 33 | (27.5%) | 136 | (16.6%) |  | 0.0039 | 1.90 | (1.22-2.95) |
| *B*44:03:02* | 1 | (0.8%) | 0 | (0%) |  | 0.0090 | - |  |
| *B*46:01:01* | 21 | (17.5%) | 68 | (8.3%) |  | 0.0014 | 2.34 | (1.37-3.98) |
| *B*48:01:01* | 6 | (5%) | 49 | (6%) |  | - | 0.82 | (0.35-1.97) |
| *B*51:01:01* | 16 | (13.3%) | 129 | (15.8%) |  | - | 0.82 | (0.47-1.43) |
| *B*51:02:01* | 3 | (2.5%) | 4 | (0.5%) |  | 0.0169 | 5.21 | (1.15-23.58) |
| *B*52:01:01* | 10 | (8.3%) | 178 | (21.8%) |  | 0.0006 | 0.33 | (0.17-0.64) |
| *B*54:01:01* | 9 | (7.5%) | 114 | (14%) |  | - | 0.50 | (0.25-1.01) |
| *B*55:02:01* | 7 | (5.8%) | 30 | (3.7%) |  | - | 1.63 | (0.7-3.79) |
| *B*56:01:01* | 3 | (2.5%) | 6 | (0.7%) |  | - | 3.47 | (0.86-14.05) |
| *B*56:03* | 1 | (0.8%) | 5 | (0.6%) |  | - | 1.36 | (0.16-11.78) |
| *B*59:01:01* | 6 | (5%) | 32 | (3.9%) |  | - | 1.29 | (0.53-3.16) |
| *B*59:04* | 1 | (0.8%) | 0 | (0%) |  | 0.0090 | - |  |
| *B*07:169* | 0 | (0%) | 1 | (0.1%) |  | - | 0.00 |  |
| *B*15:07:01* | 0 | (0%) | 8 | (1%) |  | - | 0.00 |  |
| *B*15:10:01* | 0 | (0%) | 1 | (0.1%) |  | - | 0.00 |  |
| *B*15:28* | 0 | (0%) | 2 | (0.2%) |  | - | 0.00 |  |
| *B*27:05:02* | 0 | (0%) | 1 | (0.1%) |  | - | 0.00 |  |
| *B*38:02:01* | 0 | (0%) | 4 | (0.5%) |  | - | 0.00 |  |
| *B*39:02:03* | 0 | (0%) | 1 | (0.1%) |  | - | 0.00 |  |
| *B*39:04* | 0 | (0%) | 6 | (0.7%) |  | - | 0.00 |  |
| *B*39:23* | 0 | (0%) | 2 | (0.2%) |  | - | 0.00 |  |
| *B*40:03:01* | 0 | (0%) | 10 | (1.2%) |  | - | 0.00 |  |
| *B*40:52* | 0 | (0%) | 1 | (0.1%) |  | - | 0.00 |  |
| *B*48:01:NEW* | 0 | (0%) | 1 | (0.1%) |  | - | 0.00 |  |
| *B*55:04* | 0 | (0%) | 2 | (0.2%) |  | - | 0.00 |  |
| *B*58:01:01* | 0 | (0%) | 6 | (0.7%) |  | - | 0.00 |  |
| *B*67:01:01* | 0 | (0%) | 16 | (2%) |  | - | 0.00 |  |
| *B*67:01:02* | 0 | (0%) | 3 | (0.4%) |  | - | 0.00 |  |
| *B*78:04* | 0 | (0%) | 1 | (0.1%) |  | - | 0.00 |  |
| *HLA-C* |  |  |  |  |  |  |  |  |
| *C*01:02:01* | 45 | (37.5%) | 233 | (28.5%) |  | 0.0443 | 1.50 | (1.01-2.24) |
| *C*01:03* | 1 | (0.8%) | 9 | (1.1%) |  | - | 0.75 | (0.09-6.01) |
| *C*03:03:01* | 25 | (20.8%) | 205 | (25.1%) |  | - | 0.79 | (0.49-1.25) |
| *C*03:04:01* | 38 | (31.7%) | 178 | (21.8%) |  | 0.0164 | 1.66 | (1.09-2.53) |
| *C*04:01:01* | 10 | (8.3%) | 67 | (8.2%) |  | - | 1.02 | (0.51-2.04) |
| *C*04:82* | 4 | (3.3%) | 13 | (1.6%) |  | - | 2.13 | (0.68-6.65) |
| *C*05:01:01* | 3 | (2.5%) | 7 | (0.9%) |  | - | 2.97 | (0.76-11.63) |
| *C*06:02:01* | 2 | (1.7%) | 12 | (1.5%) |  | - | 1.14 | (0.25-5.14) |
| *C*07:02:01* | 22 | (18.3%) | 216 | (26.4%) |  | - | 0.62 | (0.38-1.02) |
| *C*07:04:01* | 3 | (2.5%) | 13 | (1.6%) |  | - | 1.59 | (0.45-5.65) |
| *C*07:06* | 1 | (0.8%) | 0 | (0%) |  | 0.0090 | - |  |
| *C*08:01:01* | 9 | (7.5%) | 93 | (11.4%) |  | - | 0.63 | (0.31-1.29) |
| *C*08:03:01* | 3 | (2.5%) | 22 | (2.7%) |  | - | 0.93 | (0.27-3.14) |
| *C*08:22* | 1 | (0.8%) | 6 | (0.7%) |  | - | 1.14 | (0.14-9.52) |
| *C*12:02:02* | 11 | (9.2%) | 178 | (21.8%) |  | 0.0013 | 0.36 | (0.19-0.69) |
| *C*14:02:01* | 12 | (10%) | 94 | (11.5%) |  | - | 0.85 | (0.45-1.61) |
| *C*14:03* | 33 | (27.5%) | 136 | (16.6%) |  | 0.0039 | 1.90 | (1.22-2.95) |
| *C*15:02:01* | 8 | (6.7%) | 55 | (6.7%) |  | - | 0.99 | (0.46-2.13) |
| *C*02:02:02* | 0 | (0%) | 2 | (0.2%) |  | - | 0.00 |  |
| *C*03:02:02* | 0 | (0%) | 6 | (0.7%) |  | - | 0.00 |  |
| *C*03:03:17* | 0 | (0%) | 1 | (0.1%) |  | - | 0.00 |  |
| *C*03:04:04* | 0 | (0%) | 1 | (0.1%) |  | - | 0.00 |  |
| *C*03:03:NEW* | 0 | (0%) | 1 | (0.1%) |  | - | 0.00 |  |
| *C*12:03:01* | 0 | (0%) | 1 | (0.1%) |  | - | 0.00 |  |
| *C*14:03:NEW* | 0 | (0%) | 1 | (0.1%) |  | - | 0.00 |  |

| **Supplementary Table 3 \| Primers used for Sanger sequencing validation** | | |  |
| --- | --- | --- | --- |
| NGS typing | PCR-SSOP (Luminex) typing | Oligonucleotide | Position |
| *A*31:01:NEW* | *A*31:01* | 5'-CCGAAGGCGGTGTATGGATTG-3' | HLA-A, 5'UTR |
|  |  | 5'-CTGTCGAACCGCACGAACTG -3' | HLA-A, exon1 |
| *B*48:01:NEW* | *B*48:01* | 5'-CCCACACTTGCTTTCCTCGT-3' | HLA-B, intron5 |
|  |  | 5'-TGTGAGAGACACATCAGAGCC-3' | HLA-B, intron6 |
| *C*14:03:NEW* | *C*14:03* | 5'-AGGATTACATCGCCCTGAACG-3' | HLA-C, exon3 |
|  |  | 5'-TGATCCCATTTTCCTCCCCTC -3' | HLA-C, intron3 |
| *C*03:04:NEW* | *C*03:04* | 5'-TGTCTTCTCAGGGAAAGCAGAA-3' | HLA-C, intron4 |
|  |  | 5'-ACTGCCCTCCTAAGGTCTGTC -3' | HLA-C, intron5 |
| *A*02:15N* | *A*02:07* | 5'-TATGACTCACCACGCTGTCTC-3' | HLA-A, intron3 |
|  |  | 5'-AGAGGCTCCTGCTTTCCCTAA -3' | HLA-A, intron4 |
| *A*11:02* | *A*11:01* | 5'-TCTAAAGTCCGCACGCACC-3' | HLA-A, intron1 |
|  |  | 5'-TGAGGGGTCGTGACCTGC -3' | HLA-A, intron2 |
| *B*07:169* | *B*07:02* | 5'-GTGGTCCTAGGGTGTCCCAT-3' | HLA-B, intron3 |
|  |  | 5'-GATATGACCCCTCATCCCCCT -3' | HLA-B, intron4 |
| *B*59:04* | *B*59:01* | 5'-TTACATCGCCCTGAACGAGG-3' | HLA-B, exon3 |
|  |  | 5'-CAAGGAGGGCGACATTCTA-3' | HLA-B, intron3 |
| *C*04:82* | *C*04:01* | 5'-GGATGAGGGGTGATGTGTCTTC-3' | HLA-C, intron4 |
|  |  | 5'-TGGGGCACACTTCTACCTGG -3' | HLA-C, intron5 |
| *C*07:06* | *C*07:01* | 5'-AACCTTCCAGAAGTGGGCAG-3' | HLA-C, intron4 |
|  |  | 5'-TGTGTGGATGGTGCTTCCAG-3' | HLA-C, intron5 |
|  |  | 5'-TGTTTCCTGATCCTGCCCTG-3' | HLA-C, intron5 |
|  |  | 5'-CCACTTCAGCTCCCCAGAAT-3' | HLA-C, intron6 |
| *C*08:22* | *C*08:01* | 5'-TGTTTCCTGATCCTGCCCTG-3' | HLA-C, intron5 |
|  |  | 5'-CCACTTCAGCTCCCCAGAAT-3' | HLA-C, intron6 |
| Abbreviations: NGS, next generation sequencing ; PCR-SSOP, polymerase chain reaction sequence specific oligonucleotide probing ; UTR, untranslated region. These primers were designed according to the genomic sequences of candidate regions by Primer-BLAST (https://www.ncbi.nlm.nih.gov/tools/primer-blast/). | | | |
|  |  |  |  |
|  |  |  |  |
